# Assessment of Quality of Life in Infertility Treated Women in Poland

**DOI:** 10.3390/ijerph18084275

**Published:** 2021-04-17

**Authors:** Artur Wdowiak, Agnieszka Anusiewicz, Grzegorz Bakalczuk, Dorota Raczkiewicz, Paula Janczyk, Marta Makara-Studzińska

**Affiliations:** 1 Diagnostic Techniques Unit, Medical University of Lublin, 20-081 Lublin, Poland; wdowiakartur@gmail.com; 2 Independent Public Clinical Hospital No. 4, 20-954 Lublin, Poland; agnieszkaanusiewicz@wp.pl; 3 International Scientific Association for the Support and Development of Medical Technologies, 20-012 Lublin, Poland; grzegorz.bakalczuk@gmail.com; 4 Center of Postgraduate Medical Education, Department of Medical Statistics, School of Public Health, 01-826 Warsaw, Poland; dorota.bartosinska@gmail.com; 5 Institute of Nursing and Midwifery, Faculty of Health Sciences, Jagiellonian University Medical College, 31-501 Kraków, Poland; marta.makara-studzinska@uj.edu.pl

**Keywords:** FertiQoL, WHOQOL-BREF, assisted reproductive technologies, infertility, quality of life, intrauterine insemination, in vitro fertilization

## Abstract

The aim of this study was to assess the quality of life (QoL) of infertility treated women as it can affect the effectiveness of therapy. This cross-sectional study was conducted with Abbreviated World Health Organization Quality of Life questionnaire (WHOQOL-BREF), Fertility Quality of Life tool (FertiQoL) and an author’s questionnaire. The study included 1200 women treated for infertility without the use of assisted reproductive technology (non-ART), intrauterine insemination (IUI), or in vitro fertilization (IVF). The control group was 100 healthy women who had children. The time to conceive did not significantly differ between study groups and was 3.1–3.6 years, on average. The quality of life in the WHOQOL-BREF questionnaire data significantly differed between study groups and the control (physical domain *p* < 0.001, psychological *p* = 0.009; social *p* = 0.004; environmental *p* < 0.001). A significant effect was found in 4 FertiQoL subscales: emotional, biological, partnership, and attitude towards treatment; depending on the method of treatment. Women who received non-ART treatment evaluated their QoL in significantly more negative terms in these 4 subscales, compared to those treated with IVF. The quality of life depends on reproductive problems, methods of infertility treatment, age, place of residence, and education level. Prolongation of the duration of treatment unfavourably affects the quality of life. The quality of life of women undergoing infertility treatment differs according to the mode of work and having children from a previous relationship.

## 1. Introduction

Today, nearly every fifth couple at reproductive age experiences problems with having children, and the World Health Organization considers infertility as a social disability [1]. From the social perspective, being childless is the reason for considering a marriage as dysfunctional. In every society there is an axiological-normative system according to which its members should act. From a sociological point of view, childlessness is treated in society as a deviation, a deviation from the norm or a stigma. The knowledge of norms and stereotypes influences the fact that infertility is negatively assessed by the spouses themselves, even when they have not experienced, and do not directly experience, labelling and social reaction. The stigmatization of infertility occurs not only through the social evaluation it is subjected to, but also through the pressure to become parents to which the spouses are directly subjected by their immediate environment: relatives, friends, and acquaintances [2]. When a long-lasting treatment is unsuccessful, there appears to be frustration of a social, psychological, and existential character [3]. This fact causes deterioration of the quality of life of patients with reproductive problems. Social frustration results from an inability to fulfil the social role of rearing children. Persons with reproductive problems are under severe pressure from society and are often exposed to ridicule and criticism of the environment. Most often, the disapproval is directed towards the woman. Fear of criticism is especially pronounced in persons with a neurotic need for acceptance. Therefore, in childless women a tendency may be observed towards withdrawal and avoidance of social contacts [4].

Psychological frustration is highly related with social frustration. Self-image and self-esteem are among the most important mechanisms regulating personality. With prolonged treatment and a lack of self-confidence there occurs an intensification of excitability, anxiety, and even depressive reactions. Not having a baby feels like another frustration due to an unsatisfied need for love [3,5,6,7].

Existential frustration is the third type of frustration to which childless persons are exposed. The essential driving forces behind human activities include the need for meaning and purpose in life to strive for. Many people complain of emptiness and lack of meaning in their lives. Women, more often than men, focus on family matters and in these matters, they see the meaning of life [6].

Couples diagnosed with infertility are initially recommended for hormone tests, assessment of ovulation, assessment of the patency of the fallopian tubes, and evaluation of the semen of the male partner. Based on the results of these examinations, the physician chooses the method of infertility treatment. Sometimes this is stimulation of ovulation, intrauterine inseminations, or in vitro fertilization.

During infertility treatment, apart from biological assessment of a patient’s condition, their emotional experiences, general wellbeing, and possibilities of functioning in daily life are also evaluated. According to some reports, the emotional status exerts an effect on the chance of becoming pregnant. Considering the occurrence of interdependencies between physical, emotional, and social functioning of patients, and the degree of intensity of somatic complaints, an assessment of the quality of life is an important element of patient management. Standardized quality of life questionnaires enable the obtaining of reliable and comparable results of the assessment of the quality of life. Specific questionnaires are designed for the measurement of the quality of life of patients with a particular illness, and in the case of infertility the dedicated questionnaire is the FertiQoL [8].

The aim of the presented study was a comparison of the quality of life of women treated due to infertility with various methods, and an analysis of socio-demographic and health conditioning of the occurring differences, in order to better understand which women would need more intensive emotional and psychological support.

## 2. Materials and Methods

### 2.1. Study Groups

The study was conducted during the period from January 2017 to June 2018, at the OVUM Medical Centre and in the OVEA Obstetric—Gynaecological Office, both in Lublin, Poland.

Consent for the study was obtained from the Bioethical Committee at the Medical University in Lublin, No. KE-0254/306/2016.

The process of recruiting the study participants, both for the study group and for the control group, was carried out by directly asking all patients in the above-mentioned facilities. The study was voluntary and anonymous. Consent for participation in the study was obtained after explaining its goal and course.

The study group consisted of women who applied for treatment, or those who were already treated due to infertility. The criterion for inclusion into the study group was the diagnosis of infertility according to the WHO classification [9], which is the failure to achieve pregnancy after at least 1 year of regular unprotected intercourse. Women who reported due to both primary and secondary infertility were qualified. Additionally, male infertility was not a disqualifying factor. The participants had to be of reproductive age. Women who participated in the study were first treated using intrauterine insemination (IUI) or in vitro fertilization (IVF), without the use of assisted reproductive technology (non-ART), and the control group was with confirmed fertility. In each case, the survey was carried out 24–35 h after pharmacological induction of ovulation, whereas in the control group, it was carried out during ovulation confirmed using the urine ovulation tests. We did not analyse whether the women conceived as a result of the treatment. All data were collected using the surveys.

The control group were women who reported to check-up visits to the gynaecological consultation room. The interview method was used. It was necessary for the woman to provide information about previous pregnancy: at least one childbirth within 2–4 years prior to the study was the condition for inclusion into the study. The criterion of a minimum of 2 years after pregnancy was established, because of the need to exclude the likely effect of increased prolactin levels during breastfeeding on fertility disorders. The criterion of 4 years was adopted because it is the time during which the ovarian reserve should not be significantly reduced; therefore, it could not have a potential impact on female fertility. In addition, the time to become pregnant could not exceed 1 year. The pregnancy had to be the result of natural conception, could not be achieved by Assisted Reproductive Techniques (ART), nor could it be the result of any form of fertility treatment.

The study was carried out by the method of a diagnostic survey, using an author-constructed questionnaire, the Abbreviated World Health Organization Quality of Life questionnaire (WHOQOL-BREF) [10], and the Polish version of the Fertility Quality of Life tool (FertiQoL) [11].

The author-constructed questionnaire contained items concerning socio-demographic data, such as age, place of residence, level of education, having children, mode and type of employment, monthly net income per one person in a household, as well as time to become pregnant, and weight and height in order to calculate the body mass index. The primary version of the questionnaire was applied in a pilot study which included a sample of 30 respondents. Using the information from the pilot study and the respondents’ comments, some questions were modified, others were removed, and few new questions were added, and also the set of multiple-choice answers was supplemented.

### 2.2. Abbreviated World Health Organization Quality of Life Questionnaire (WHOQOL-BREF)

The WHOQOL-BREF questionnaire [10] contains 26 items. First two questions are of general nature. Question 1 measures overall perception of the quality of life, question 2 measures overall perception of health. The remaining items concern four domains: physical health, psychological health, social relationships, and environment. The replies to each question are provided according to the 5-point scale, from 1 to 5 scores. The scoring of the questions has a positive direction, i.e., the higher the number of scores, the higher the quality of life, except for questions: 3, 4, and 26, the higher values of which evidence lower quality of life. Therefore, scores of these items are then reversed into positive scores. The scoring for domains is established by calculating a mean score for questions belonging to a given domain. Then the result for each domain is multiplied by 4, which makes them comparable with the results obtained using the WHOQOL-100 questionnaire. Raw results may be converted into transformed scores within the range from 4–20 or 0–100. The Cronbach’s alpha coefficient for the Polish version of the WHOQOL-BREF questionnaire is equal 0.80 for the physical subscale, 0.78 for the psychological subscale, 0.63 for the social subscale, and 0.78 for the environmental subscale. The Cronbach’s alpha coefficient for the whole WHOQOL-BREF questionnaire is 0.92 for the healthy, and 0.95 for ill persons, which provides high reliability of the research instrument applied.

### 2.3. Fertility Quality of Life Tool (FertiQoL)

The FertiQoL tool [8] is used to investigate the effect of fertility problems on the quality of life, as well as an approach to treatment and its effect on the quality of life of patients treated due to infertility.

This questionnaire contains 36 closed questions. The first two questions, marked with letters A and B, concern the general state of health and satisfaction with the quality of life, and are considered neither in the total scoring nor in any of the subscales.

Replies to each question are scored according to the 5-point scale, from 1 to 5 scores. Scoring of the majority of questions has a positive direction, i.e., the higher the number of scores, the higher the quality of life, except for the questions: Q4, 11, 15, 21, 14, and T2 and T5, which have a negative direction, i.e., their higher values evidence a lower quality of life. Therefore, their scores should be reversed.

The first and main part of the questionnaire consists of 4 core subscales: emotional, relational, mind/body, and social. Questions Q4, 7, 8, 9, 16, and 23 concern the emotional sphere and show the effect of negative emotions on the quality of life (jealousy, regret, sadness, and depression). Items Q1, 2, 3, 12, 18, and 24 pertain to the mind/body sphere and demonstrate the effect of fertility disorders on physical and cognitive health, as well as behaviour. Questions Q6, 11, 15, 19, 20, and 21 concern the relational sphere and show the effect of fertility disorders on marriage and partnership. Items Q5, 10, 13, 14, 17, and 22 concern the social sphere and demonstrate how social relations have been affected by problems related with fertility.

The second part of the FertiQoL questionnaire concerns treatment and consists of two subscales: treatment environment, and treatment tolerability. Items T2, T5, T7, T8, T9, and T10 are questions from the subscale treatment environment, which demonstrate what the quality and availability of treatment is and their effect on the quality of life. Items T1, T3, T4, and T6 are questions from the subscale treatment tolerability, which show to what extent medical services provided for infertile patients affect the quality of life of these patients.

Based on the results from six subscales of the questionnaire, three joint results are calculated: assessment of the quality of life (the first—core part of the questionnaire), assessment of treatment (the second part of the questionnaire), and total assessment of the quality of life and treatment.

The results for the subscales are calculated as follows: raw scores are calculated by summing all items that belong to the subscale or total scale. Subsequently, to compute scaled scores the raw score is multiplied by 25/k, where k is the number of items in each subscale. The scaled scores are obtained within the range from 0 to 100. The higher the result in the subscales and total score, the better is the quality of life.

### 2.4. Statistical Methods

Statistical analyses were conducted using SPSS software. Minimum and maximum values mean (M) and standard deviation (SD) were estimated for continuous variables, as well as absolute numbers (n) and percentages (%) of the occurrence of items for categorical variables. The following statistical tests were used:

- Pearson’s chi-square test to compare the categorical variables between study groups;
- F test for analysis of variance to compare continuous variables between study groups and the least significant difference test was used as post hoc test.

Multiple regression was used to correlate total score of FertiQoL with characteristics in three study groups according to infertility treatment method.

The significance level was assumed to be 0.05.

## 3. Results

### 3.1. Respondents’ Characteristics

Since the recruitment process was through convenience sampling and participation was voluntary, all women who were willing to participate were included in the study. We have lost 23 out of 1223 questionnaires due to incomplete data (3 lost in non-ART, 9 lost in IUI, and 11 lost in the IVF group).

The study included 1200 women who were treated due to infertility with various methods:

- 400 women were treated without the use of assisted reproductive technology techniques (non-ART),
- 400 were treated using the intrauterine insemination method (IUI),
- 400 women were treated with the use of in vitro fertilization (IVF).

The control group were 100 women with confirmed fertility, i.e., who had children (2–4 years after childbirth).

The mean age of the examined women who had children was 33.7 years, similar to those treated using IVF (mean age 33.2), whereas infertile women treated without the use of assisted reproductive technology techniques (non-ART) and those treated using IUI were one year younger, on average. The largest number of respondents from all groups were urban inhabitants, had a higher level of education, normal body weight, and had non-manual and permanent employment. As many as 78% of the women who received non-ART treatment, 86% of those treated using IUI, and 87% using IVF had no children. Time to become pregnant ranged from 1 to 10 years, approximately 3.5 years, on average (Table 1).

### 3.2. Comparison of the Quality of Life (WHOQOL-BREF) between Respondents with Infertility and Those Who Had Children

**Figure 1:**
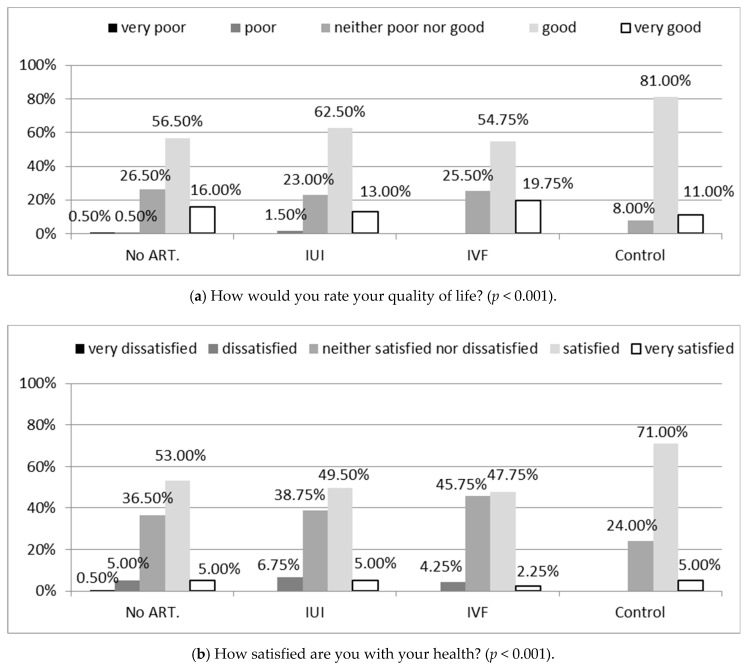
General quality of life (**a**) and health satisfaction (**b**) according to WHOQOL-BREF in study groups. Chi-square test was used. no ART.—Women treated for infertility without the use of assisted reproductive technology; IUI—intrauterine insemination; IVF—in vitro fertilization; WHOQOL-BREF—Abbreviated World Health Organization Quality of Life questionnaire.

The highest percentage of women from the group with confirmed fertility evaluated their quality of life as good or very good (92%), whereas women in the group treated due to infertility significantly less commonly assessed their overall quality of life as good (72.5% of those who received non-ART treatment, 75.5% IUI, and 74.5% IVF), while more frequently as neither poor nor good (*p* < 0.001), (Figure 1a).

Similar regularities concerned overall satisfaction with health (*p* < 0.001), (Figure 1b).

Women in all the examined groups evaluated their physical health lower than other domains, and slightly better assessed their psychological health, whereas they provided the highest evaluations concerning social relations and environment (Table 2). Women treated for infertility evaluated their psychological health and social relationships significantly lower than those from the control group (*p* < 0.05). Physical health and environment were assessed significantly lower by women treated with IUI or IVF than by women from the control group (*p* < 0.05). However, no significant differences in physical health and environment were found between women treated with non-ART and the control group (*p* = 0.636 and *p* = 0.293, respectively).

### 3.3. Quality of Life According to the FertiQoL versus Method of Infertility Treatment

**Figure 2:**
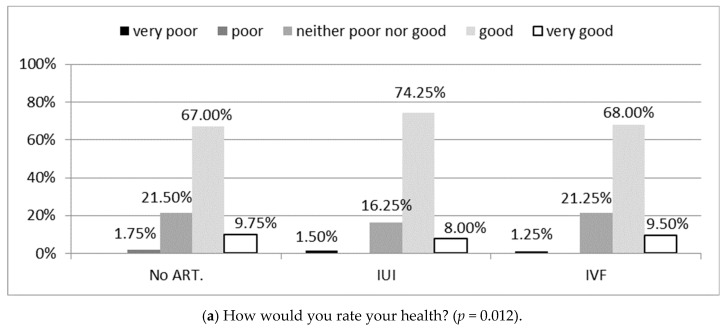

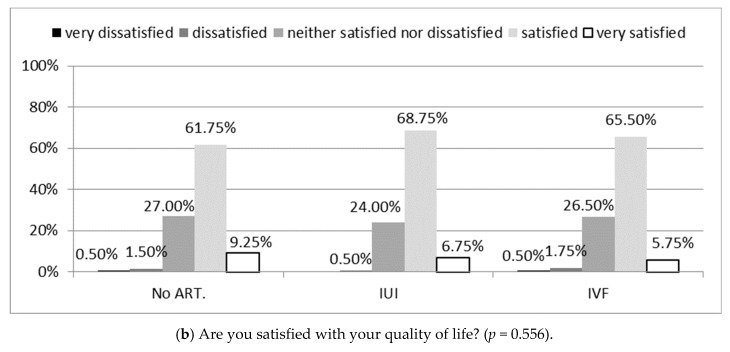
General health (**a**) and quality of life satisfaction (**b**) according to FertiQoL in study groups. Chi-square test was used. no ART.—Women treated for infertility without the use of assisted reproductive technology; IUI—intrauterine insemination; IVF—in vitro fertilization; FertiQol—Fertility Quality of Life tool.

Self-assessment of the state of health by the women in the study significantly differed between the 3 groups of infertility treatment: non-ART, IUI, and IVF (*p* = 0.012). Women treated with IUI evaluated their health significantly better than those who received non-ART treatment or IVF (*p* = 0.012), (Figure 2a). In turn, the assessments of satisfaction with the quality of life did not significantly differ between women who received non-ART treatment, IUI, or IVF (*p* = 0.556), (Figure 2b).

The three methods of infertility treatment exerted a significant effect on the overall quality of life and health. Total FertiQoL scores ranged from 64.3, on average, in women who received non-ART treatment, followed by 65.2 in those treated using IUI, and 66 in women treated using IVF (Table 3). Women from all groups evaluated treatment more positively than Core FertiQoL. The emotional domain was evaluated the lowest by the IUI group, and most positively by the IVF group, with the non-ART group in between (*p* = 0.001). Relational domain was assessed more positively by the IUI group, compared to the non-ART and IVF groups (*p* = 0.022).

### 3.4. Correlations between Quality of Life According to FertiQoL and Characteristics of the Study Groups

According to the FertiQoL, the quality of life was significantly lower among better educated women and those who tried to become pregnant for a longer time using all 3 analysed methods (Table 4). A positive correlation was observed between age and assessment of the quality of life in the group of women who received non-ART or IVF treatment. The evaluations of the quality of life provided by women treated using IUI and IVF were significantly lower among women living in rural areas, than among urban women, and lower among women who received non-ART treatment with a higher BMI.

Evaluations of the quality of life were significantly correlated with the level of education of women who received non-ART treatment or treated with IUI, whereas no significant correlation was observed in women treated with IVF. The lowest quality of life was found in women who received non-ART treatment or IVF, those working in shifts or not in normalized employment compared to permanent employment. No correlations were observed in the quality of life with having children, mode of employment, and income per capita in women treated using the 3 analysed methods.

## 4. Discussion

FertiQoL is an international questionnaire constructed specially for the measurement of the quality of life of patients treated due to infertility. This survey was validated for many countries worldwide, which allowed the comparison of results obtained by other researchers with our results. A study concerning Europe, conducted in Germany, by Sexty et al. [12] showed considerably higher results in all categories of the FertiQoL in Germany in comparison to our results in a Polish population. In another study carried out in Germany by Herrmann et al. using the WHOQOL [13], the researchers obtained a higher result in physical and environmental domains, whereas the results obtained in psychological and social domains were on a similar level. A lower assessment of the quality of life in our study may result from the fact that in the Lublin Province, where the study was carried out, the economic conditions of daily life are worse, compared to western Poland and West European countries. Moreover, it is worth mentioning that in Germany the state contributes to the costs of infertility treatment. However, in Poland the costs are mostly, or entirely, borne by the couple undertaking the treatment. Pastoralist programs over the past years have offered marginal financial support only for diagnostics of selected couples. This may explain the lower social and physical scores for Polish women, as they have to ensure solvency with a considerable burden of costs of infertility treatment. The results of a study conducted in the Netherlands by Aarts et al. [14] using FertiQoL clearly demonstrated that the results of the FertiQoL obtained in Polish patients were evidently lower concerning overall, biological, and social subscales, while with respect to the relation and emotional subscales they were close to the results obtained in the Dutch study. The Netherlands is a predominantly secular country. In Poland, the dominant Catholic faith does not recognize assisted reproduction methods, such as IVF, which may increase feelings of social stigma among couples undertaking this type of treatment [15].

However, the results obtained in the presented study are on a higher level for women treated for infertility, compared to a similar group of women treated due to polycystic ovary syndrome (PCOS) in the study by Rzońca et al. carried out in the same province [16]. Women with PCOS are characterized by a lower level of acceptance of the disease, which may exert an effect on the result obtained by them using the WHOQOL [17]. Moreover, the results of the quality of life of women with endometriosis show even lower QoL indicators, compared to those with PCOS examined in the same area of Poland [18], which indicates, that women undertaking infertility treatment are in a better self-reported state than women treated with other gynaecological conditions.

An Asian study conducted by Hsu et al. [19] in the Taiwanese population demonstrated that in the biological, partnership, and treatment categories the results obtained in Taiwan were clearly lower than those obtained in Poland, while the results pertaining to the social and emotional categories were similar in both populations. In a study by Hee-Jun et al. [20] carried out in Korea, the researchers also obtained lower results in all categories of the FertiQoL, compared to the results obtained in our study. Maroufizadeh et al. [21] examined the population of Iranian patients and obtained results which differed from our results exclusively in the subscale treatment and were clearly lower. Undoubtedly, the cause of these differences may be culturally conditioned in association with the practiced religion and the resulting role of a woman, financial issues, and problems with availability of treatment. In an original study by Boivin et al. [8] conducted in the USA, Australia, Canada, and the United Kingdom, the results obtained in all FertiQoL categories compared were lower than those obtained in the presented study. The differences observed between individual reports may have multifactorial conditioning. Mainly, it is likely that the passage of time since 2011 and improved living conditions could affect the overall QoL scores. Improved availability and effectiveness of infertility treatment, in addition to public awareness, may explain the better results in all categories.

In the present study we tried to evaluate which factors may have potential influence on QoL in Polish women undergoing different forms of treatment. As the results show, the age of a woman seems to be an important factor. In their study, Aarts et al. [14] obtained a positive correlation between age and an overall result of the FertiQoL. The respondents’ age showed a positive relationship also in the biological, emotional, and social subscales. Sexty et al. [12] also obtained a positive relationship between the age of the examined women and the emotional and biological subscales. In the presented study, the evaluations of the quality of life positively correlated with the age of women who received non-ART treatment and those undergoing IVF, which seems to be consistent with the abovementioned results. It may be presumed that the issues related with age, education, and the type of occupation performed may be translated into financial situation and, therefore, may shape the quality of life.

However, in the study of Iranian women, Maroufizadeh et al. obtained different results and did not observe a straightforward relationship between age and any of the FertiQoL subscales for women undergoing IVF [21]. Their results showed a positive effect on the emotional and mind/body subscale score, whereas it had a negative effect on the relational subscale. In the present study we did not undertake as detailed analysis, which may be considered as a study limitation and potentially be in line with the results obtained. Probably, after an extended analysis, we could indicate the reason why age did not prove to be a protective factor among the IUI women while it was protective for IVF and non-ART treatment.

A study by Keramat et al. [22] conducted among Iranian patients showed a relationship and better results in the emotional subscale of the FertiQoL in respondents who were occupationally active, and did not have any history of previous infertility treatment; in the biological subscale higher results were obtained by patients with higher education, were occupationally active, and without history of previous treatment; in the partnership subscale higher results were obtained by patients who had better socio-economic status and possessed a smaller number of children; whereas no relationships were observed in the social subscale. In turn, in our study, the evaluations of the quality of life and treatment positively correlated with patients’ age (except IUI) and were also significantly lower in women living in rural areas, compared to urban inhabitants in the non-ART and IVF groups, and close to a significant difference for the IUI group. In addition, the evaluations of the quality of life positively correlated in all groups with the level of education of the examined women, while the assessment of treatment did not show such a relationship, as is also seen in Aarts et al. research [14]. The results obtained in this respect may be, in all issues, associated with the economic status, which is higher in urban than rural areas, and among older, better educated respondents. This is in line with results of other researchers in terms of economic status, education level, and age [23,24].

Moreover, women treated using IVF who had children from the current relationship evaluated their quality of life and treatment in significantly more negative terms, than those who had no children or had children from a previous relationship. The relationship observed is difficult to explain. This may possibly be related with the fact that women are more satisfied at the beginning of the relationship but while trying to conceive again with the same partner the relationship begins to fade. Possibly, this is due to the relationship confirmed in our study that the evaluations of the quality of life negatively correlated with the number of persons in women’s households. Raque-Bogdan et al. also suggest the increased stress in women with secondary infertility due to accelerated fertility-related social concerns [25].

Despite the abovementioned findings, some limitations need to be underlined. Firstly, it has not been analysed why there are certain differences between study groups (non-ART, IUI, and IVF treatment). The additional analysis with detailed enumeration of which FertiQoL categories show differences could provide insight into why some characteristics are influential for some groups while not for the others.

Finally, only some variables were assessed in this study, while others would require consideration. This study could be improved by conducting a comparison of QoL with anxiety and depression syndromes, which are proven to be coexisting with lower QoL scores [14], and it potentially may explain the differences presented between groups.

The results of the presented study demonstrated the difficult life situation of patients treated due to infertility in Poland. Their quality of life is worse than in West European countries. This may be associated with the lack of reimbursement for the procedures of assisted reproductive technology in Poland. Considering the scale of the problem of infertility, a need arises for the provision of support for these persons in their difficult life situation. Undoubtedly, further studies of this problem would be advisable, with consideration of the present epidemiological situation.

## 5. Conclusions

1. Reproductive problems can influence the quality of life of affected women.
2. Prolongation of the time of infertility treatment negatively affects the quality of life of women undergoing therapy.
3. The quality of life of patients treated due to infertility depends on their age, place of residence, and education level.
4. Intellectual work, on permanent basis, without burden of hazardous factors, exerts a favourable effect on the evaluation of the quality of life of women treated due to infertility.

## Figures and Tables

**Table 1:** Study groups’ characteristics.

Variable, Parameter	IU or Category	Infertility Treated Women			Control Group (N = 100)	Comparison between Groups *p*
		Non-ART (N = 400)	IUI (N = 400)	IVF (N = 400)		
Age, Min–Max, M ± SD	years	20–43, 32.7 ± 4.4	22–44, 32.4 ± 4.5	24–42, 33.2 ± 4.0	27–43, 33.7 ± 4.4	0.007
Place of residence, n (%)	city	162 (40.50)	162 (40.50)	158 (39.50)	51 (51.00)	0.050
	town	101 (25.25)	91 (22.75)	100 (25.00)	21 (21.00)	
	rural area	137 (34.25)	147 (36.75)	142 (35.50)	28 (28.00)	
Level of education, n (%)	basic vocational	25 (6.25)	5 (1.25)	30 (7.50)	0 (0.00)	<0.001
	secondary	99 (24.75)	96 (24.00)	94 (23.50)	19 (19.00)	
	tertiary	276 (69.00)	299 (74.75)	276 (69.00)	81 (81.00)	
BMI, Min–Max, M ± SD	kg/m^2^	15.4–37.2, 24.2 ± 4.1	16.4–38.9, 24.0 ± 4.7	15.2–37.8, 24.5 ± 4.3	17.7–37.1, 25.2 ± 5.5	0.083
BMI, n (%)	underweight	20 (5.00)	31 (7.75)	15 (3.75)	4 (4.00)	0.007
	normal weight	249 (62.25)	234 (58.50)	257 (64.25)	50 (50.00)	
	overweight	90 (22.50)	85 (21.25)	82 (20.50)	22 (22.00)	
	obesity	41 (10.25)	50 (12.50)	46 (11.50)	24 (24.00)	
Having children, n (%)	yes, from the current relationship	39 (9.75)	40 (10.00)	23 (5.75)	100 (100.00)	<0.001 *
	yes, from a previous relationship	48 (12.00)	18 (4.50)	28 (7.00)		
	no	313 (78.25)	342 (85.50)	349 (87.25)	0 (0.00)	
Time to get pregnant, Min–Max, M ± SD	years	1–10, 3.4 ± 2.1	1–9, 3.2 ± 2.1	1–10, 3.6 ± 2.0	-	0.090
Mode of employment, n (%)	manual	91 (22.75)	78 (19.50)	97 (24.25)	14 (14.00)	0.281
	non-manual	195 (48.75)	215 (53.75)	196 (49.00)	57 (57.00)	
	mixed	114 (28.50)	107 (26.75)	107 (26.75)	29 (29.00)	
Type of employment, n (%)	constant	183 (45.75)	226 (56.50)	187 (46.75)	58 (58.00)	0.010
	shift	129 (32.25)	89 (22.25)	123 (30.75)	22 (22.00)	
	not normalized	88 (22.00)	85 (21.25)	90 (22.50)	20 (20.00)	
Monthly net income per 1 person (thousand PLN), n (%)	below 1	38 (9.50)	27 (6.75)	29 (7.25)	2 (2.00)	0.001
	1–1.5	103 (25.75)	87 (21.75)	94 (23.50)	19 (19.00)	
	1.5–2	148 (37.00)	128 (32.00)	169 (42.25)	40 (40.00)	
	above 2	111 (27.75)	158 (39.50)	108 (27.00)	39 (39.00)	

* without control group, Chi-square test for categorical variables or F test for quantitative variables. M—Mean; SD—Standard Deviation; BMI—Body Mass Index; PLN—Polish currency—Polish złoty; non-ART—women treated for infertility without the use of assisted reproductive technology; IUI—intrauterine insemination; IVF—in vitro fertilization.

**Table 2:** WHOQOL-BREF scores in study groups.

Domain	Infertility Treated Women			Control Group (N = 100)	Comparison between Groups *p*	*p* for Post Hoc Tests					
	Non-ART (N = 400)	IUI (N = 400)	IVF (N = 400)			Non-ART vs. Control	IUI vs. Control	IVF Control	Non-ART vs. IUI	Non-ART vs. IVF	IUI vs. IVF
Physical health, M ± SD	56.6 ± 8.2	54.8 ± 8.3	54.9 ± 7.4	57.1 ± 9.3	<0.001	0.636	0.012	0.018	0.001	0.003	0.803
Psychological, M ± SD	66.6 ± 8.5	66.2 ± 8.5	66.1 ± 9.5	69.1 ± 9.2	0.009	0.012	0.003	0.002	0.517	0.425	0.881
Social relationships, M ± SD	71.7 ± 11.8	71.9 ± 12.2	70.3 ± 13.4	75.6 ± 13.0	0.004	0.006	0.008	0.001	0.832	0.109	0.070
Environment, M ± SD	69.8 ± 7.6	68.6 ± 8.6	68.5 ± 8.9	70.8 ± 8.8	<0.001	0.293	0.021	0.018	0.047	0.038	0.927

F test for analysis of variance was used. The least significant difference test was used as a post hoc test. M—Mean; SD—Standard Deviation; non-ART—women treated for infertility without the use of assisted reproductive technology; IUI—intrauterine insemination; IVF—in vitro fertilization; WHOQOL-BREF—Abbreviated World Health Organization Quality of Life questionnaire.

**Table 3:** FertiQoL scores in study groups.

Domain	Infertility Treated Women			Comparison between Groups *p*	*p* for Post hoc Tests		
	Non-ART (N = 400)	IUI (N = 400)	IVF (N = 400)		Non-ART vs. IUI	Non-ART vs. IVF	IUI vs. IVF
Total FertiQoL, M ± SD	64.3 ± 12.1	65.2 ± 11.4	66.1 ± 12.7	0.109	0.272	0.035	0.315
Core FertiQoL, M ± SD	61.9 ± 14.1	62.8 ± 13.4	63.8 ± 14.5	0.152	0.385	0.053	0.282
Emotional, M ± SD	55.4 ± 18.9	53.8 ± 19.5	58.8 ± 17.8	0.001	0.212	0.010	<0.001
Mind/body, M ± SD	59.9 ± 18.6	60.4 ± 17.6	62.5 ± 18.4	0.105	0.722	0.047	0.102
Relational, M ± SD	71.8 ± 17.3	74.8 ± 15.6	72.3 ± 16.6	0.022	0.009	0.617	0.035
Social, M ± SD	60.5 ± 17.1	62.1 ± 16.9	61.6 ± 16.6	0.398	0.187	0.350	0.701
Treatment FertiQoL, M ± SD	66.7 ± 12.8	67.7 ± 12.0	68.3 ± 13.0	0.163	0.249	0.059	0.460
Environment, M ± SD	66.5 ± 15.1	67.2 ± 14.1	68.0 ± 13.8	0.332	0.531	0.139	0.394
Tolerability, M ± SD	66.8 ± 18.0	68.2 ± 17.3	68.6 ± 17.3	0.289	0.252	0.131	0.715

F test for analysis of variance was used. The least significant difference test was used as a post hoc test. M—Mean; SD—Standard Deviation; non-ART—women treated for infertility without the use of assisted reproductive technology; IUI—intrauterine insemination; IVF—in vitro fertilization; FertiQoL—Fertility Quality of Life tool.

**Table 4:** Multivariable regression analysis results for total FertiQoL scores versus characteristics in study groups.

Covariate	IU or Category	Infertility Treated Women					
		Non-ART (N = 400)		IUI (N = 400)		IVF (N = 400)	
		b	*p*	b	*p*	b	*p*
Age	years	0.51	<0.001	0.23	0.074	0.37	0.020
Place of residence	city	0.46	0.585	2.43	0.002	2.34	0.009
	town	1.26	0.171	0.60	0.508	0.55	0.561
	rural area	reference					
Level of education	basic vocational or secondary	2.36	0.002	3.78	<0.001	2.63	0.001
	tertiary	reference					
BMI	kg/m^2^	−0.33	0.036	0.18	0.139	−0.29	0.071
Having children	yes	0.01	0.998	0.08	0.919	−1.52	0.106
	no	reference					
Time to get pregnant	years	−0.68	0.022	−0.58	0.043	−0.96	0.004
Mode of employment	non-manual	reference					
	manual or mixed	−0.08	0.923	−0.86	0.266	0.39	0.630
Type of employment	constant	1.51	0.031	1.24	0.056	1.64	0.023
	shift or not normalized	reference					
Monthly net income per 1 person (thousand PLN), n (%)	below 1.5	reference					
	1.5–2	−0.27	0.761	−1.33	0.129	−1.26	0.175
	above 2	0.06	0.942	−0.20	0.804	0.57	0.494

General linear regression was used. b—regression slope term i.e., mean change in total FertiQoL scores per unit of a covariate. M—Mean; SD—Standard Deviation; BMI—Body Mass Index; PLN—Polish currency—Polish złoty; non-ART—women treated for infertility without the use of assisted reproductive technology; IUI—intrauterine insemination; IVF—in vitro fertilization; FertiQoL—Fertility Quality of Life tool.

## Data Availability

The datasets generated during and/or analysed during the current study are available from the corresponding author on reasonable request.
